# The disconnect between classical biostatistics and the biological data mining community

**DOI:** 10.1186/1756-0381-6-12

**Published:** 2013-07-24

**Authors:** James D Malley, Jason H Moore

**Affiliations:** 1 Center for Information Technology, The National Institutes of Health, Bethesda, MD, United States of America; 2 Departments of Genetics and Community and Family Medicine, Institute for Quantitative Biomedical Sciences, The Geisel School of Medicine, Dartmouth College, One Medical Center Dr, Lebanon, NH, 03756, United States of America

## 

Statistics departments and journals still strongly emphasize a very narrow range of topics and methods and techniques, all driven by a tiny handful of results, many dating from the 1930s. Those methods may well have been good and amazing and quite appropriate for the available computing, known mathematical facts, and data of their day. Hence the common list of assumptions: normal distributions and very small parametric models and linearity and independent features. But the usual claims for these anchoring assumptions are accurate—when precisely true—but more often just irrelevant: data is rarely normal, model misspecification is always at work, features are highly entangled with functionally mysterious interactions, and multiple scientifically plausible models may all fit the data equally well.

Thus, linearity is largely a convenience for the researcher for downstream interpretation—obviously an important task—but typically with no justified scientific grounding. Similarly for parametric models with a tiny handful of parameters and tidy inclusion of only multiplicative interactions. Assuming normality for error terms (a dreadful misnaming by statisticians: Nature doesn't make errors, statisticians do) is fine when valid, and then familiar big statistical theorems can apply. And linear correlation as a measure of association assumes, well, that the data (X, Y) is linear in Y given X. But in Big Data or even doll-house data, it can be hard or impossible to evaluate the assumption.

But this brings us to the alternatives that are currently widely ignored by the statistical community. These are important mathematical and statistical developments over the last forty years that make no appearance in many statistics classes and journals. These methods are broad extensions of familiar results but are just as often complex combinatorial arguments, and all seem invisible to the statistical community while being conventional in the machine learning community. Two classics in the field are: [1] for nonparametric classification, and [2] for nonparametric regression. Both provide background at a nearly conversational level along with fully rigorous treatment of the deep theory. In more detail, both introduce and motivate the Vapnik-Chervonenkis results from the 1970s, and numerous more recent generalizations, on statistical complexity and empirical error minimization; See also [3]. Intensive further work has shown how practical these deep results can be, in for example, in easily and optimally setting up a Random Forest analysis on a data set of any size in any sense, a thousand subjects and five predictors or a hundred subjects and two million SNPs; See also [4]; Chapter 2].

The situation, this disconnect between deep theory and practical methods, is unsettling. Hard-fought battles have already been won and then anatomized in the machine learning literature, but the older zombie methods persist in the statistics literature and teaching.

It is important for readers to be appraised of these developments and given the chance to implement them. They also need the chance to see machine learning predictive models in a larger, less constrained world. Too often small and well-worn technical toolkits serve the purpose of declaring certain key problems as Unanswerable and thus as Ignorable. This is less than ideal. We all need methods that are both reasonably easy to apply and potentially insightful. So the distinction is between a comfortable, well-traveled road and an energizing if uncertain trail. But novel and big problems should compel novel solutions and not persistence of historical artifact. Community sanctioned or self imposed toolkits wall us off from methods with unexpected benefits even as they challenge us. And both these outcomes are good things.
